# Alterações no Perfil dos Pacientes atendidos no Pronto Socorro durante o Surto de COVID-19 em um Hospital Geral Especializado em Tratamento Cardiovascular no Brasil

**DOI:** 10.36660/abc.20200595

**Published:** 2021-01-27

**Authors:** Thiago Veiga Jardim, Flavio Veiga Jardim, Luciana Muniz Veiga Jardim, Juliana Tranjan Coragem, Cristovão Fernandes Castro, Guilherme Moreira Firmino, Paulo Cesar B. Veiga Jardim

**Affiliations:** 1 Universidade Federal de Goiás Programa de Pós-Graduação em Ciências da Saúde GoiâniaGO Brasil Universidade Federal de Goiás - Programa de Pós-Graduação em Ciências da Saúde, Goiânia, GO - Brasil; 2 Universidade Federal de Goiás Liga de Hipertensão Arterial GoiâniaGO Brasil Universidade Federal de Goiás - Liga de Hipertensão Arterial, Goiânia, GO - Brasil; 3 Hospital do Coração de Goiás GoiâniaGO Brasil Hospital do Coração de Goiás, Goiânia, GO - Brasil; 4 Universidade Federal de Goiás GoiâniaGO Brasil Universidade Federal de Goiás – Cardiologia, Goiânia, GO – Brasil

**Keywords:** Doenças Cardiovasculares, COVID-19, SARS-CoV-2, Coronavírus, Pandemia, Síndrome Respiratória Aguda, Hospitalização, Hospital Público, Epidemiologia

## Introdução

A doença do novo coronavírus (COVID-19) é causada pelo vírus da síndrome respiratória aguda grave coronavírus 2 ( *severe acute respiratory syndrome-coronavirus* -2, SARS-CoV-2).^1^ Em 11 de março de 2020, a COVID-19 foi declarada uma pandemia pela Organização Mundial de Saúde, e o primeiro caso no Brasil foi relatado no final de fevereiro.^2^

Dada a ausência de tratamento específico e os altos índices de morbimortalidade da COVID-19, especialmente nos grupos de risco, medidas extraordinárias de saúde pública vêm sendo implementadas em todo o mundo.^1^ A estratégia tradicional de saúde pública contra surtos de doenças, que consiste em isolamento, quarentena, distancimento social e confinamento, foi implementada em vários países e teve papel fundamental na prevenção da disseminação da doença.^3^

Desde que o primeiro caso de COVID-19 foi relatado no Brasil, além de medidas de distanciamento social, iniciou-se uma grande campanha para que os pacientes evitassem procurar atendimento médico em emergências, exceto em caso de extrema necessidade. A maioria das ações da campanha ocorreu nas mídias sociais, na mídia tradicional, e em relatórios governamentais.^4 , 5^ Essas ações eram justificadas pela preocupante disseminação da COVID-19 nas emergências e pelo hábito da população brasileira de procurar as emergências como uma alternativa ao atendimento regular com um médico da atenção básica.^6^

O número de pacientes em todo o país que buscaram atendimento médico nas emergências por motivos que não síndrome respiratória aguda diminuiu de forma significativa, especialmente depois da implementação das medidas de distanciamento social.^7 , 8^ Apesar dessas alterações, não existem dados científicos sobre o real impacto do surto de COVID-19 nas emergências do Brasil. A fim de preencher essa lacuna de conhecimento, comparamos as características sociodemográficas e clínicas dos pacientes que bucaram o setor de emergência antes e depois do surto de COVID-19 no Brasil.

## Métodos

Realizamos um estudo retrospectivo unicêntrico que avaliou os prontuários de todos os pacientes consecutivos que buscaram atendimento no setor de emergência de um hospital geral privado especializado em cuidados cardiovasculares. Essa instituição está localizada na capital de um estado da região Centro-Oeste do Brasil. Os dados dos pacientes atendidos antes da implementação da quarentena na cidade foram comparados aos daqueles atendidos após a implementação. O estudo foi aprovado pelo Comitê de Ética da instituição e, visto que não seria utilizado nenhum dado de identificação dos pacientes, não foi necessária a assinatura de um formulário de consentimento.

O número médio de pacientes atendidos no setor de emergência da instituição em 2019 foi de 1500/mês. Visto que as medidas de distanciamento foram oficialmente implementadas em 16 de março de 2010 na cidade em que está localizada a instituição, por meio de uma resolução do estado, decidimos comparar os dados dos 2 meses após a implementação da quarentena (durante – 16 de março de 2020 a 16 de maio de 2020) com os do mesmo período do ano anterior (antes – 16 de março de 2019 a 16 de maio de 2019).

As variáveis avaliadas foram: número de pacientes, idade, sexo, cidade de residência, plano de saúde, motivo para buscar atendimento médico, tempo gasto no setor de emergência, era um funcionário do hospital, solicitou afastamento por doença, recebeu medicamento, realizou exames laboratoriais ou de imagem, realizou eletrocardiograma (ECG), recebeu alta da emergência, necessitou de internação hospital, necessitou de internação na unidade de terapia intensiva (UTI).

A descrição detalhada dos métodos é apresentada no material suplementar.

## Resultados

**Figura 1:**
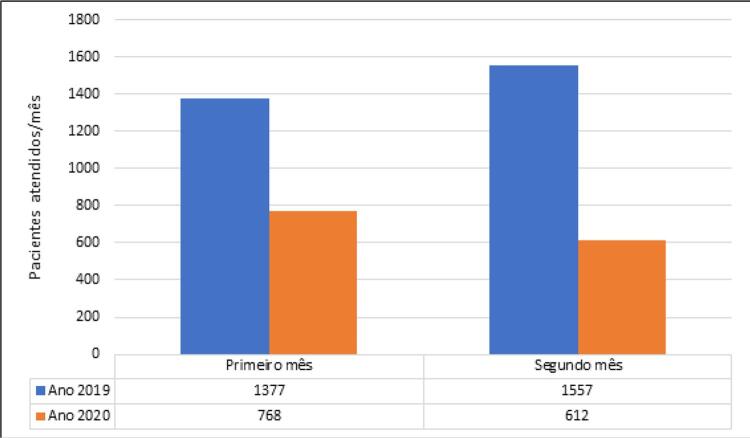
– Comparação do número de pacientes atendidos no setor de emergência/mês em um mesmo período de tempo no ano anterior e durante o distanciamento social motivado pela COVID-19. Primeiro mês – de 16 de março a 15 de abril. Segundo mês – de 16 de abril a 16 de maio.

Durante os 2 meses avaliados em 2019 (antes da COVID-19), o número total de pacientes atendidos no setor de emergência foi de 2934. Esse número diminuiu para 1380 nos mesmos meses em 2020 (durante a COVID-19), o que representou uma redução de 57% no número total de pacientes atendidos. A Figura 1 demonstra o número de pacientes atendidos/mês durante o período de tempo analisado.

As características sociodemográficas dos pacientes atendidos no setor de emergência antes e durante a crise da COVID-19 estão detalhadas na tabela S1 (material suplementar). A idade média dos pacientes diminuiu, assim como o percentual de pacientes ≥ 60 anos e de pacientes atendidos no setor de emergência provenientes de outras cidades que não Goiânia. O percentual de funcionários do hospital e de pacientes sem plano de saúde aumentou durante o surto de COVID-19.

Ao se avaliar as diferenças clínicas entre os pacientes e tratamentos antes e durante o surto de COVID-19, houve alterações significativas em quase todas as variáveis. O número de pacientes classificados como urgentes na triagem aumentou, assim como o tempo gasto no setor de emergência. Houve também um aumento do número de todos os procedimentos diagnósticos realizados no setor de emergência (ECG, exames laboratoriais e de imagem), enquanto o uso de medicamentos diminuiu. A quantidade de pacientes que necessitaram de interação hospitalar aumentou, especialmente daqueles que necessitaram de internação na UTI. Ao se comparar os diagnósticos mais comuns, houve uma diminuição nos casos de gastroenterite infecciosa e de dengue. Por outro lado, os casos de transtorno de ansiedade aumentaram, assim como os de síndrome respiratória viral. Não houve alteração no percentual de casos de doença cardiovascular, embora tenha havido uma redução de 49,6% no número absoluto de casos. A Tabela 1 apresenta um resumo de todos esses achados.

**Tabela 1:** – Diferenças clínicas relativas a pacientes e tratamentos antes e durante o surto de COVID-19 no setor de emergência de um hospital terciário privado brasileiro

Variável	Antes da COVID-19	Durante a COVID-19	Valor de p
**n**	2934	1380	
**Classificado como urgente na triagem**	491 (16,7%)	276 (20,0%)	0,009
Tempo gasto no SE* (minutos)	277,8 (222,6)	194,7 (140,0)	<0,001
**Solicitou afastamento por doença**	146 (5,0%)	177 (12,8%)	<0,001
Medicamento no SE*	1958 (66,7%)	846 (61,3%)	<0,001
Teste laboratorial no SE*	311 (10,6%)	612 (44,3%)	<0,001
Eletrocardiograma no SE*	897 (30,6%)	533 (38,6%)	<0,001
Exame de imagem no SE*	812 (27,7%)	502 (36,4%)	<0,001
Alta do SE*	2617 (89,2%)	1132 (82,0%)	<0,001
**Internação hospitalar**	236 (8,0%)	138 (10,0%)	0,033
**Internação na UTI†**	81 (2,8%)	110 (8,0%)	<0,001
**Doença cardiovascular**	474 (16,2%)	235 (17,0%)	0,470
**Gastroenterite infecciosa/ colite**	160 (5,5%)	22 (1,6%)	<0,001
**Dengue**	240 (8,2%)	18 (1,3%)	<0,001
**Transtornos de ansiedade**	115 (3,9%)	110 (8,0%)	<0,001
**Doenças do sistema geniturinário**	92 (3,1%)	36 (2,6%)	0,340
**Doenças do sistema digestivo **	62 (2,1%)	34 (2,5%)	0,470
**Doenças do sistema musculoesquelético e do tecido conjuntivo**	102 (3,5%)	56 (4,1%)	0,340
**Síndrome respiratória viral**	21 (0,7%)	203 (14,7%)	<0,001

Valores expresso como média (± desvio padrão) ou n (%). *SE – setor de emergência; †UTI – unidade de terapia intensiva.

Além disso, a tabela S2 (material suplementar) detalha as diferenças sociodemográficas e clínicas entre os pacientes com e sem síndrome respiratória viral. As diferenças mais significativas em favor daqueles que não apresentaram síndrome respiratória viral foram observadas no percentual de pacientes ≥ 60 anos, que foram classificados como urgentes na triagem, que necessitaram de medicamento e que realizaram ECG no setor de emergência. Por outro lado, as diferenças mais significativas em favor dos pacientes com síndrome respiratória viral foram observadas no percentual de paciente que eram funcionários do hospital, que realizaram exames de imagem e que solicitaram afastamento por doença.

## Discussão

Alterações significativas no número de pacientes atendidos nas emergências de todo o mundo durante o surto de COVID-19 foram relada em cartas ao editor, pontos de vista e documentos não cientificos. Entretanto, segundo nosso conhecimento, este é o primeiro estudo científico o apresentar os resultados dessas alterações com dados da vida real. De fato, nosso estudo observou uma redução significativa do número de pacientes no setor de emergência, a qual alcançou 57%. Ocorreram alterações na frequência de diagnósticos, além de diferenças no atendimento prestado a esses pacientes.

A comparação dos 2 meses após a implementação das medidas oficiais de distanciamento social motivadas pela COVID-19 com o período equivalente do ano anterior baseou-se nas diferenças sazonais dos pacientes atendidos nas emergências. Na região onde o estudo foi realizado, arboviroses, especialmente a dengue, são condições altamente prevalentes no período analisado.^9^ Diante disso, acreditamos que nossa comparação é a mais confiável e eficaz na prevenção de vieses.

Houve uma redução de 49,6% no número absoluto de pacientes com doenças cardiovasculares atendidos no setor de emergência. Um estudo italiano obteve resultados semelhantes ao avaliar apenas as internações hospitalares por infarto agudo do miocárdio durante uma semana em comparação a uma semana equivalente em 2019.^10^ Outro estudo, realizado nos EUA, observou um redução de até 48% nas taxas semanal de hospitalização por infarto agudo do miocárdio durante o período da pandemia de COVID-19.^11^ Embora a redução do número absoluto observada em nosso estudo seja semelhante à de outros dados internationais, não houve alteração no percentual de pacientes com doenças cardiovasculares atendidos no setor de emergência durante o surto de COVID-19.

Um aspecto interessante dos resultados aqui apresentados é o aumento do percentual de pacientes com transtornos de ansiedade atendidos no setor de emergência durante a pandemia de COVID-19.^12^ Esse achado é corroborado por várias publicações que avaliaram a COVID-19 e também as medidas de distanciamento social e o seu impacto na saúde mental da população.^13 - 15^

As características clínicas dos casos suspeitos/confirmados de COVID-19 podem ser observadas nos nossos resultados ao se comparar os pacientes com e sem síndrome respiratória viral. Primeiramente, o atendimento a esses pacientes demanda mais tempo, conforme demonstrado pelo aumento significativo do tempo gasto no setor de emergência. Visto que se trata de uma doença altamente contagiosa, são solicitados mais afastamentos por doença. O número de pacientes atendidos que trabalhavam no hospital também aumentou, sugerindo uma alta prevalência da doença em profissionais da saúde, conforme já relatado anteriormente.^16^ Por fim, o aumento do número de pacientes que necessitaram de internação na UTI demonstra a gravidade da doença.^17^

As potenciais limitações deste estudo devem ser mencionadas. Em primeiro lugar, trata-se de um estudo unicêntrico realizado na capital de um estado no qual o número de casos de COVID-19 era baixo se comparado ao de outras capitais do Brasil. Em segundo lugar, selecionamos os diangósticos mais comuns, de acordo com a definição do médico assistente do setor de emergência, deixando assim de investigar algumas doenças. Por último, as comorbidades dos pacientes não foram relatadas, porque essa informação não estava disponível no banco de dados utilizado no estudo.

É importante ressaltar que a coleta de dados durante uma emergência de saúde pública é extremamente desafiadora. Todos os esforços estão voltados para a pandemia, tanto no cuidado aos pacientes quanto na preocupação dos prestadores de cuidados de saúde em se contaminarem. Ainda assim, quanto mais dados científicos forem disponibizados, melhores serão os cuidados para tratar a COVID-19 e todas as outras doenças que estão afetando os pacientes nesses tempos tão difíceis. Outro aspecto importante é o fato de que este é um estudo observacional em que foram descritas as alterações nas características dos pacientes. Sendo assim, não é possível estabelecer uma relação causa-efeito precisa.

^*^Material suplementar

Para informação adicional, por favor, clique aqui
