# Supplementary material for: Alterações no Perfil dos Pacientes atendidos no Pronto Socorro durante o Surto de COVID-19 em um Hospital Geral Especializado em Tratamento Cardiovascular no Brasil
Source: Arq Bras Cardiol. 2021 Jan 27;116(1):140–3. [Article in Portuguese] doi: 10.36660/abc.20200595 (PMC8159515; doi:10.36660/abc.20200595)
Supplement: Translation

# Translation: Changes in the Profile of Emergency Room Patients during the COVID-19 Outbreak in a General Hospital Specialized in Cardiovascular Care in Brazil

**Authors:** Thiago Veiga Jardim, Flavio Veiga Jardim, Luciana Muniz Veiga Jardim, Juliana Tranjan Coragem, Cristovão Fernandes Castro, Guilherme Moreira Firmino, Paulo Cesar B. Veiga Jardim

**Affiliations:** 1 Brazil Universidade Federal de Goiás - Programa de Pós-Graduação em Ciências da Saúde, Goiânia, GO - Brazil; 2 Brazil Universidade Federal de Goiás - Liga de Hipertensão Arterial, Goiânia, GO - Brazil; 3 Brazil Hospital do Coração de Goiás, Goiânia, GO - Brazil; 4 Brazil Universidade Federal de Goiás – Cardiologia, Goiânia, GO – Brazil

**Keywords:** Cardiovascular Diseases, COVID-19, SARS-CoV-2, Coronavirus, Pandemics, Acute Respiratory Syndrome, Hospitalization, Public Hospital, Epidemiology

## Introduction

The coronavirus disease 2019 (COVID-19) is caused by the severe acute respiratory syndrome coronavirus 2 (SARS-CoV-2).^1^On March 11, 2020, COVID-19 was declared a pandemic by the World Health Organization, and the first case was reported in Brazil by the end of February.^2^

Given the absence of specific treatment and the high morbidity and mortality of COVID-19, particularly in high-risk groups, extraordinary public health measures have been implemented worldwide.^1^Considering public health, the traditional outbreak response strategy of isolation, quarantine, social distancing, and community containment has been implemented in multiple countries and has played an important role in preventing disease spread.^3^

Since the first COVID-19 case was reported in Brazil, in addition to social distancing measures, a massive campaign has been implemented to prevent patients from seeking medical care at emergency rooms (ER) unless extremely necessary. Most campaign actions took place on social media, traditional media, and government reports.^4 , 5^These actions were justified by the worrisome COVID-19 spread in ERs and the habit of the Brazilian population of seeking ER care as an alternative to regular care with primary care physicians.^6^

The number of patients around the country seeking medical assistance in ERs for reasons other than acute respiratory syndromes has decreased significantly, particularly after the implementation of social distancing measures.^7 , 8^Despite these changes, there is a lack of scientific data on the real impact of the COVID-19 outbreak on ERs in Brazil. Aiming to address this knowledge gap, we compared the sociodemographic and clinical characteristics of patients seeking ERs before and after the onset of the COVID-19 outbreak in Brazil.

## Methods

We conducted a retrospective single-center study assessing the medical records of all consecutive patients who sought medical care in an ER of a private general hospital specialized in cardiovascular care. This facility is located in a state capital of Brazil’s Central-West region. We compared data of patients treated before the implementation of quarantine measures in the city and those treated afterwards. The study was approved by the institution’s Ethics Committee and as no patient identification data were to be used, a consent form was not required.

The mean number of patients treated monthly in the institution’s ER in 2019 was 1500. Since social distancing measures were officially implemented on March 16, 2020 by a state resolution, we decided to compare data referring to the 2 months after quarantine implementation (March 16, 2020 to May 16, 2020) with the same period of the previous year (March 16, 2019 to May 16, 2019).

The assessed variables were: number of patients, age, sex, city of residency, health insurance, reason for seeking medical assistance, and time spent in the ER; we also evaluated whether the patient was a hospital employee, required sick leave, received medication, underwent any laboratory or imaging tests, underwent an electrocardiogram (EKG), was discharged from the ER, required hospital admission, or required admission to an intensive care unit (ICU).

Detailed descriptions of the methods are provided on the Supplemental Material.

## Results

During the 2 assessed months of 2019 (pre-COVID-19), the total number of patients treated at the ER was 2934. This number decreased to 1380 in the same months of 2020 (during COVID-19), which translates into a 57% reduction in the total number of treated patients. The number of patients treated per month during the studied time frame is shown on Figure 1 .

**Figure 1:** – Emergency room patients treated per month in the same time frame of the previous year and during COVID-19 social distancing. First month – from March 16 to April 15. Second month – from April 16 to May 16.

The sociodemographic characteristics of patients treated at the ER pre- and during the COVID-19 crisis are shown on Table S1 (Supplemental Material). Their mean age was decreased, as well as the percentage of patients aged ≥ 60 years and coming from cities other than Goiânia. The proportion of hospital employees and of patients with no health insurance increased during the COVID-19 outbreak.

When comparing the clinical characteristics of patients and treatments pre- and during the COVID-19 outbreak, we observed that almost all variables changed significantly. The number of urgent triage classifications increased, and so did the time spent by patients at the ER. The number of diagnostic procedures performed at the ER (electrocardiographies, laboratory and image tests) increased, while medication use decreased. Patients requiring hospital admission increased, particularly those requiring ICU admission. When comparing the most common diagnoses, there was a decrease in infectious gastroenteritis and dengue fever cases. Conversely, the number of patients with anxiety disorders and respiratory viral syndromes increased. No changes were seen on the proportion of cardiovascular diseases in relation to other diagnoses, although a 49.6% absolute reduction in their cases was observed. A summary of these findings is presented on Table 1 .

**Table 1:** – Clinical aspects of patients and treatments before and during the COVID-19 outbreak in an emergency room of a Brazilian private tertiary hospital

Variables	pre-COVID-19	During COVID-19	p-value
**n**	2934	1380	
**Triaged as urgent**	491 (16.7%)	276 (20.0%)	0.009
Time spent at ER* (minutes)	277.8 (222.6)	194.7 (140.0)	< 0.001
**Required sick leave**	146 (5.0%)	177 (12.8%)	< 0.001
Received medication on ER*	1958 (66.7%)	846 (61.3%)	< 0.001
Laboratory test on ER*	311 (10.6%)	612 (44.3%)	< 0.001
Electrocardiography on ER*	897 (30.6%)	533 (38.6%)	< 0.001
Image examination on ER*	812 (27.7%)	502 (36.4%)	< 0.001
Discharged from ER*	2617 (89.2%)	1132 (82.0%)	< 0.001
**Hospital admission**	236 (8.0%)	138 (10.0%)	0.033
**ICU† admission**	81 (2.8%)	110 (8.0%)	< 0.001
**Cardiovascular disease**	474 (16.2%)	235 (17.0%)	0.470
**Infectious gastroenteritis / colitis**	160 (5.5%)	22 (1.6%)	< 0.001
**Dengue fever**	240 (8.2%)	18 (1.3%)	< 0.001
**Anxiety disorders**	115 (3.9%)	110 (8.0%)	< 0.001
**Genitourinary diseases**	92 (3.1%)	36 (2.6%)	0.340
**Gastrointestinal diseases**	62 (2.1%)	34 (2.5%)	0.470
**Musculoskeletal and connective tissue diseases**	102 (3.5%)	56 (4.1%)	0.340
**Respiratory viral syndromes**	21 (0.7%)	203 (14.7%)	<0.001

Values given as means (± standard deviation) or n (%). *ER: emergency room; †ICU: intensive care unit.

Additionally, on Table S2 (Supplemental Material), the sociodemographic and clinical differences between patients with or without respiratory viral syndromes were compared. The most significant differences towards patients without respiratory viral syndromes were in the proportion of patients aged ≥ 60 years, triaged as urgent, who required medication, or underwent electrocardiography at the ER. On the other hand, the percentages of patients who were hospital employees, underwent imaging tests, or required sick leaves were the most significantly different and higher in those with respiratory viral syndromes.

## Discussion

A significant change in the number of patients treated at ERs worldwide during the COVID-19 outbreak has been reported by letters to editors, points of view, and non-scientific documents. Nevertheless, to our knowledge, this is the first scientific study presenting real-life results of these changes. In our study, we observed a significant reduction in the number of patients cared for at the ER, reaching a 57% decrease. Changes in frequencies of different diagnoses also happened, as well as in the care given to the patients.

The comparison between the 2 months following official COVID-19 social distancing measures and the same period of the previous year was based on seasonal differences observed in patients treated at ERs. In the Brazilian region where the study was conducted, arboviruses, particularly dengue fever, have a high prevalence during the assessed months.^9^Therefore, we believe that our method of comparison is the most reliable and effective for avoiding bias.

We observed a 49.6% absolute reduction in the number of patients with cardiovascular diseases treated at the ER. An Italian study found similar results when assessing only hospital admissions for acute myocardial infarction over a period of one week in comparison with the same week of 2019.^10^Another study, conducted in the USA, found that weekly hospitalization rates for acute myocardial infarction decreased by up to 48% during the COVID-19 period.^11^Although the absolute reduction found in our study was similar to other international data, we found no changes in the relative percentage of patients with cardiovascular diseases treated at the ER during the COVID-19 outbreak.

An interesting aspect of the results presented here is the increase in the percentage of patients with anxiety disorders being treated at the ER during the COVID-19 pandemic.^12^This finding is supported by various publications that assessed COVID-19, social distancing measures, and the impact on the population’s mental health.^13 - 15^

Clinical features of suspected/confirmed COVID-19 cases can be seen in our results when comparing patients with and without respiratory viral syndromes. Firstly, the treatment of these patients is time-consuming, which was indicated by a significant increase in time spent at the ER. Since this is a highly contagious disease, patients required more sick leaves. The number of treated patients who were hospital workers also increased, suggesting a high prevalence of COVID-19 in health care professionals, as previously reported.^16^Finally, the higher number of patients requiring ICU admission indicated disease severity.^17^

Potential limitations of this study need to be acknowledged. This was a single-center study conducted in the capital of a state in which the number of COVID-19 cases was low when compared to other state capitals in Brazil. Secondly, we selected only the most common diagnoses defined by the attending ER physician, which left some diseases uninvestigated. Finally, the patients’ comorbidities were not reported, since this information was not available on the database used in this study.

It is important to highlight that data collection during a public health emergency is extremely challenging. All efforts were targeted at the pandemic; not only on patient care, but also on the worrisome possibility of health care providers being infected. As more scientific data becomes available, health care teams will be able to provide better care for patients with COVID-19 and other diseases in these difficult times. Another important aspect is the fact that this is an observational study that described changes on patients’ features, thus not being accurate for establishing cause-effect relationships.

*Supplemental Materials

For additional information, please click here .
